# Cloning, Expression, and Characterization of Prophenoloxidases from Asian Corn Borer,* Ostrinia furnacalis* (Gunée)

**DOI:** 10.1155/2016/1781803

**Published:** 2016-12-18

**Authors:** Shasha Zhang, Fang Hong, He Song, Lei Wang, Qizhi Liu, Chunju An

**Affiliations:** Department of Entomology, China Agricultural University, Beijing 100193, China

## Abstract

Insect phenoloxidase (PO) belongs to the type 3 copper protein family and possesses oxidoreductase activities. PO is typically synthesized as a zymogen called prophenoloxidase (PPO) and requires the proteolytic activation to function. We here cloned full-length cDNA for 3 previously unidentified PPOs, which we named OfPPO1a, OfPPO1b, and OfPPO3, from Asian corn borer,* Ostrinia furnacalis* (Gunée), in addition to the previously known OfPPO2. These conceptual PPOs and OfPPO2 all contain two common copper-binding regions, two potential proteolytic activation sites, a plausible thiol-ester site, and a conserved C-terminal region but lack a secretion signal peptide sequence at the N-terminus.* O. furnacalis* PPOs were highly similar to other insect PPOs (42% to 79% identity) and clustered well with other lepidopteran PPOs. RT-PCR assay showed the transcripts of the 4 OfPPOs were all detected at the highest level in hemocytes and at the increased amounts after exposure to infection by bacteria and fungi. Additionally, we established an* Escherichia coli* (*E. coli*) expression system to produce recombinant* O. furnacalis* PPO proteins for future use in investigating their functions. These insights could provide valuable information for better understanding the activation and functioning mechanisms of* O. furnacalis* PPOs.

## 1. Introduction

Reactive oxygen species (ROS) are chemically reactive molecules that contain oxygen [1, 2]. Endogenous ROS are produced as byproducts during aerobic respiration or by oxidation reactions catalyzed by metal and oxidoreductase enzymes such as phenoloxidases (POs) [3, 4]. POs, together with tyrosinases, catechol oxidase, and hemocyanin, belong to the type 3 copper protein family, all of which have an active site composed of two histidine-coordinated copper atoms [5, 6]. PO possesses both monophenol monooxygenase activity (EC 1.14.18.1, tyrosine, dihydroxyphenylalanine, oxygen, and oxidoreductase) and* o*-diphenoloxidase activity (EC 1.10.3.1,* o*-diphenol, oxygen, and oxidoreductase) [7, 8]. Therefore, POs can catalyze the hydroxylation of monophenols to* o*-diphenols and subsequent oxidation of* o*-diphenols to corresponding highly reactive* o*-quinones [9]. PO-generated quinones act as cross-linkers for wound healing or polymerize to form melanin capsules around the invading parasites and parasitoids [10, 11] or interact with oxygen to form ROS to kill microbial pathogens directly [4, 12].

Although type 3 copper proteins are distributed among almost all organisms, POs are mainly found in arthropods including insects [6, 13]. In insects, the number of PO genes ranges from ten in the mosquito* Aedes aegypti* [14] to just one in the honeybee* Apis mellifera* [15]. In most Lepidoptera, there are two PO genes [16]. For example, two PO sequences have been reported in the silkworm* Bombyx mori* [17], the beetle* Holotrichia diomphalia* [18], and the tobacco hornworm* Manduca sexta* [19]. However, three transcripts for potential PO were identified in the transcriptome of Asian corn borer,* Ostrinia furnacalis* (Gunée) [20].

PO is predominantly synthesized in hemocytes as a zymogen called prophenoloxidase (PPO) and released into hemolymph (insect blood) to perform functions [16]. However, all arthropod PPOs studied so far lack a signal peptide for secretion into hemolymph [11, 16]. PPO is believed to be released after rupture of hemocytes [21, 22]. Then inactive PPO is converted into active PO form by proteolytic cleavage in hemolymph [23–25]. The cleavage site in PPO has been experimentally confirmed in several insect species, including ^51^Arg-Phe^52^ in* B. mori *PPO [26], ^51^Arg-Phe^52^ in* M. sexta* PPO [27, 28], and ^50^Arg-Phe^51^ followed by the second cleavage at ^162^Arg-Ala^163^ in* H. diomphalia *PPO [23, 24]. In vitro, PPO can also be activated without proteolytic cleavage by certain chemicals such as ethanol or detergents (e.g., sodium dodecyl sulfate (SDS), cetylpyridinium chloride (CPC)) [29, 30].

The activation of PPO is necessary for PO to function. A model has generally been accepted for PPO activation in insects: upon pathogenic infections or physical injuries, a serine protease cascade is triggered to activate the prophenoloxidase-activating protease and prophenoloxidase-activating protease then cleaves PPO at a conserved peptide bond to produce PO [11, 20]. This process involves multiple proteins, including pattern recognition proteins [31], a series of serine proteases [32], serine protease homologs [33], and serine protease inhibitors [34].

The Asian corn borer,* O. furnacalis* (Gunée) (Lepidoptera: Crambidae), is an important insect pest in Asia, causing serious damage to corn, sorghum, and millet [35]. However, knowledge about PPO in* O. furnacalis* is very incomplete. Feng et al. purified a PPO from* O. furnacalis* hemolymph [36] and cloned its encoding cDNA [37]. Our previous work identified three transcripts for potential PPOs in the transcriptome of this pest [20]. Among these, only one, designated as* OfPPO2*, contained a complete open reading frame [20, 37]. In this study, we cloned a full-length nucleotide sequence for another two* O. furnacalis PPOs*, which we called* OfPPO1* and* OfPPO3* (GenBank: KX437621).* OfPPO1* from the transcriptome is actually an assembly mosaic, consisting of two variant PPO sequences,* OfPPO1a* (GenBank: KX452359) and* OfPPO1b* (GenBank: KX437622), which are 86.82% identical in amino acid sequences. We investigated the expression profiles of these 4* PPOs* in different developmental stages, in different tissues, and upon the infection of different microorganisms. Additionally, we performed the recombinant expression for these 4 proteins using a prokaryotic expression system.

## 2. Materials and Methods

### 2.1. Insect Rearing

*O. furnacalis* larvae were reared on an artificial diet at 28°C under a relative humidity of 70–90% and a photoperiod of 16 h light and 8 h darkness [38].

### 2.2. Molecular Cloning of* O. furnacalis* PPO cDNAs

**Figure 1:**
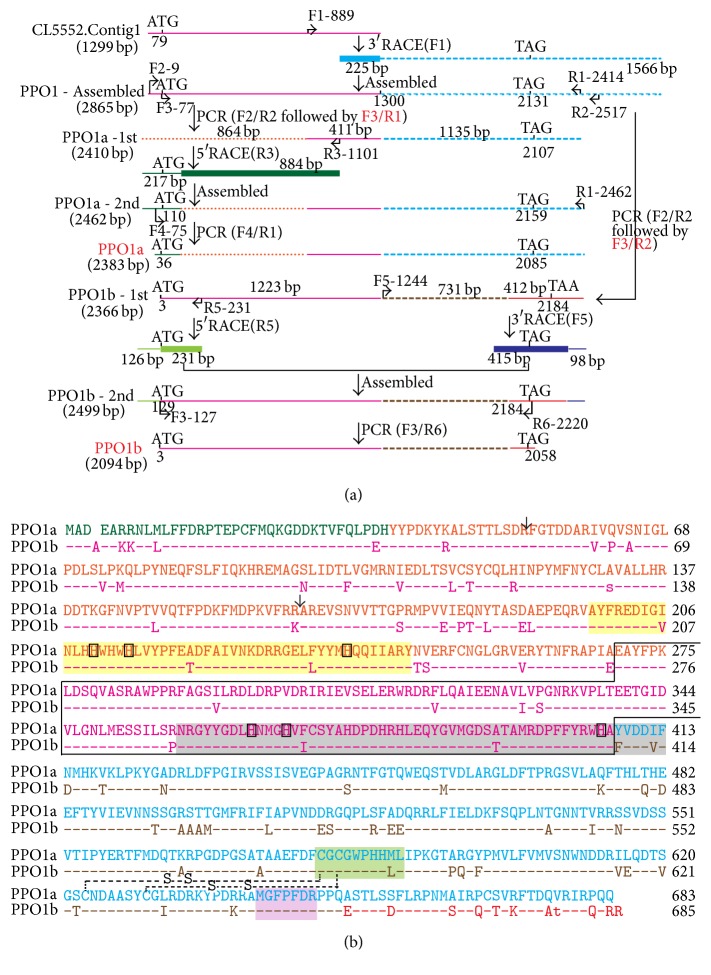
Cloning of* OfPPO1a* and* OfPPO1b*. (a) Schematic demonstration of the process for cloning full-length* OfPPO1a *(NCBI accession number: KX452359) and* OfPPO1b *(NCBI accession number: KX437622). The fragments with identical nucleotide sequences are illustrated in lines with the same color. The specific primers are labeled with the name followed by its position in the located fragment and indicated by the arrows above or below the lines. The predicted start codon and stop codon are indicated at the corresponding positions. (b) Comparisons and sequence analysis of deduced OfPPO1a and OfPPO1b. The deduced amino acid residues are shown in the same color scheme as in part (a). Identical residues in PPO1a and PPO1b are substituted by hyphens in PPO1b. Regions common in PPO1a and PPO1b are indicated by an irregular rectangle. The predicted proteolytic cleavage bonds were shown in red and marked with black arrow. The potential copper binding site A (CuA), copper binding site B (CuB), the putative thiol ester sites, and a conserved motif at the C-terminus were shaded in yellow, grey, green, and purple, respectively. The six histidine residues absolutely conserved within the CuA and CuB were in the square boxes. Two predicted disulfide linkages are represented by dashed lines.

Three transcripts for potential* PPOs *(CL5552.contig1, CL997.contig1, and Unigene28348) were previously identified from* O. furnacalis* transcriptome [20]. Among these, CL997.contig1 was a complete cDNA sequence, but another two transcripts (1,299-bp CL5552.contig1 and 1,152-bp Unigene28348) were incomplete. On the basis of these two fragments, primers (see Table  S1 in Supplementary Material available at http://dx.doi.org/10.1155/2016/1781803) were designed for 5′ and 3′ rapid amplification of cDNA ends (RACE) to obtain the remaining sequences. The 5′ and 3′ RACE reactions were performed according to the manufacturer's instructions for its RACE kit (Takara, Biotechnology (Dalian) Co., Ltd., China) with cDNA from the whole body of fifth-instar* O. furnacalis* larvae collected 24 h after injection with 3 *μ*L of* Micrococcus luteus* (3 *μ*g/*μ*L). Some modifications were made to obtain the 3′-end of CL5552.contig1. Briefly, after the first-round 3′ RACE reaction using primer F1 (Table  S1), a 1,566-bp fragment with 225 bp at 5′-end covered by CL5552.contig1 was obtained. It was then assembled into a 2,865-bp cDNA sequence (“PPO1-assembled” in Figure 1(a)) combined with CL5552.contig1 (Figure 1(a)). However, two variant PCR products, which are both inconsistent with expected “PPO1-assembled,” were amplified with selected primer pairs F3/R1 or F3/R2 (Figure 1(a), Table  S1). They were designated as “PPO1a-1st” and “PPO1b-1st,” respectively. The second-round 5′ and 3′ RACE reactions were performed for “PPO1a-1st” and “PPO1b-1st” following the manufacturer's instructions (Figure 1(a)). The resulting products were cloned and sequenced. After assembling, primers (Table  S1) were designed to amplify the full-length cDNA encompassing the entire reading frame using the same larval cDNA for RACE. The final PCR products (“PPO1a” and “PPO1b”) were cloned into pMD19-T vector (Takara, Biotechnology (Dalian) Co., Ltd, China), and the nucleotide sequences were confirmed by DNA sequencing.

### 2.3. Sequence Analysis of* O. furnacalis* PPOs

We carried out a series of sequence analyses for* OfPPO1a*,* OfPPO1b*,* OfPPO2*, and* OfPPO3*. The deduced amino acid sequences were obtained by using the Translate tool provided by the Swiss Institute Bioinformatics. Analysis of deduced amino acid sequences, including prediction of signal peptide, molecular weight, and isoelectric point, was executed in the EXPASY (Expert Protein Analysis System) proteomics server (http://www.expasy.org/). Characteristic domains or motifs were identified using DNAssist PROSITE profile database (http://prosite.expasy.org/scanprosite/).

To investigate the phylogenetic relationship between* O. furnacalis *PPOs and other arthropod PPOs, “prophenoloxidase” was used as a keyword to search the nonredundant database from the National Center for Biotechnology Information (NCBI; https://www.ncbi.nlm.nih.gov/). The retrieved PPO sequences were aligned with* O. furnacalis *PPO sequences using the Clustal W program. Phylogenetic trees were constructed by the neighbor-joining method using MEGA Version 5 software [39]. For neighbor-joining method, gaps were treated as characters, and statistical analysis was performed using the bootstrap method with 1000 replicates.

### 2.4. Reverse Transcriptase- (RT-) PCR Analysis of the Expression Profiles of* O. furnacalis* PPOs

To investigate the changes of* O. furnacalis PPO *transcript levels in several developmental stages, total RNA samples were individually prepared (*n* = 5) from three different stages including egg, larvae, and pupa using TRNzol Reagent (TIANGEN, Biotech (Beijing) Co., Ltd., China). 1 *μ*g of RNA samples equally from 5 individual RNA samples in each stage was treated with DNase I (TIANGEN, Biotech (Beijing) Co., Ltd., China) and converted into first-strand cDNA from an oligo (dT) primer following the instructions for QuantScriptRT Kit (TIANGEN, Biotech (Beijing) Co., Ltd, China). The cDNA products independently from 3 biological replicates were diluted 10-fold for use as template in RT-PCR experiments. Specific primers were designed and listed in Table  S1.* O. furnacalis* ribosomal protein L8 (*rpL8*) was used as an internal standard to adjust the template amounts in preliminary PCR experiments. The thermal cycling conditions were 94°C for 2 min and then 28 cycles of 94°C for 30 s, 58°C for 30 s, and 72°C for 30 s followed by incubation at 72°C for 10 min. The PCR products were separated by electrophoresis on a 1.5% agarose gel.

To determine the expression patterns of* O. furnacalis PPOs* in different tissues, total RNA samples were isolated separately from combined heads, midguts, fat bodies, and hemocytes from 20-day-zero fifth-instar larvae. The synthesis of first-strand cDNA and RT-PCR analysis was performed as described above.

To check the expression of* O. furnacalis PPOs* after different microbial injections, day one fifth-instar larvae from the same batch were injected into the hemocele with 3 *μ*L of sterile water containing formalin-killed* Escherichia coli* DH5*α* (2 × 10^5^ cells/*μ*L), dried* M. luteus* (3 *μ*g/*μ*L),* Beauveria bassiana* suspension (2 × 10^5^ conidia/*μ*L,* B. bassiana* conidia suspension was prepared as described previously [38]), or sterile water as a control. After 20 h (10 h for* B. bassiana* treatment), five larvae were collected from the challenged group and 5 from the control group, and total RNA samples were prepared from each larva. The following RT-PCR analysis was conducted as described above.

### 2.5. Production of Recombinant* O. furnacalis* PPO Proteins

To express recombinant* O. furnacalis *PPOs in* E. coli*, the expression plasmids were constructed via FastCloning as described by Li et al. [40]. Briefly, the vector pET28a (Novagen, Germany) and the coding region of mature OfPPO1a, OfPPO1b, OfPPO2, and OfPPO3 were amplified by PCR, respectively, using specific primers listed in Table  S1. The forward primer for vector amplification (5′-CACCACCACCACCACCACTGAGAT-3′) matched to the 3′ side of the polylinker region of expression vector pET28a. The reverse primer for vector amplification (5′-GTGATGATGATGATGATGGCTGCT-3′) was reversely complementary to the 5′ side of the polylinker. The primers for* OfPPO* amplification had specific sequences and additional 15 nucleotides overlapping with the vector ends. The PCR products containing amplified vector and each* OfPPO* was mixed and digested with* Dpn*I (Takara, Biotechnology (Dalian) Co., Ltd., China) for 1 h at 37°C. The digested mixture was then directly transformed into competent* E. coli* DH5*α* cells. After sequence verification, the resulting plasmids encoding correct OfPPOs were further transformed into* E. coli* strain BL21 (DE3) cells and plated onto Luria Bertani (LB) agar with kanamycin (0.05 mg/mL) for selection. For the coexpression of two OfPPOs, the constructed plasmids encoding OfPPO1a or OfPPO1b were individually transformed to* E. coli* BL21 (DE3) cells which already contained plasmids encoding OfPPO2 or OfPPO3, named as OfPPO1a and OfPPO2 (OfPPO1a/2), OfPPO1a and OfPPO3 (OfPPO1a/3), OfPPO1b and OfPPO2 (OfPPO1b/2), and OfPPO1b and OfPPO3 (OfPPO1b/3). Then single colony was picked and inoculated into 7 mL of LB medium with kanamycin (0.05 mg/mL) and incubated overnight at 37°C with constant shaking. The following day, 2 mL of the overnight cultures was inoculated into 100 mL of LB with kanamycin (0.05 mg/mL). When OD_600_ of the culture reached 0.7, isopropyl thio-*β*-D-galactoside (IPTG) was added to a final concentration of 0.1 mM, and the recombinant PPO proteins were expressed for 12 h at 18°C. The bacteria were harvested by centrifugation at 10,000 g for 15 min and resuspended in lysis buffer (50 mM sodium phosphate, 300 mM NaCl, 10 mM imidazole, pH 8.0) to a uniform OD_600_ to ensure equal protein loading. Cells were lysed by sonication and then centrifuged at 10,000 g for 30 min. The cleared supernatant was recorded as soluble fraction (which contained the soluble recombinant PPOs) while the cell debris was recorded as insoluble fraction (which contained the insoluble recombinant PPOs).

### 2.6. SDS-Polyacrylamide Gel Electrophoresis (SDS-PAGE) and Immunoblot Analysis

For SDS-PAGE, protein samples were treated with 2 × SDS sample buffer containing Dithiothreitol (DTT) at 95°C for 5 min and then separated by 10% SDS-PAGE. Proteins were detected by staining with Coomassie brilliant blue. For immunoblot analysis, proteins were transferred onto a nitrocellulose membrane and detected with mouse anti-His (1 : 2,000) as primary antibody. Antibody binding was visualized using alkaline phosphatase-conjugated goat anti-mouse or anti-rabbit IgG (1 : 1,500) and 5-bromo-4-chloro-3-indolyl phosphate/nitro blue tetrazolium (BCIP/NBT) staining buffer containing 165 *μ*g/mL BCIP and 330 *μ*g/mL NBT in 100 mM Tris (pH 9.5), 150 mM NaCl, and 5 mM MgCl_2_.

### 2.7. Activity Assay of Recombinant* O. furnacalis* PPO Proteins

To check the PO activity of recombinant proteins, a convenient way was utilized as described by Li et al. [30]. Briefly, Cu^2+^ was also added into the culture to a final concentration of 0.5 mM upon the addition of IPTG. Following the recombinant expression as described above, 1 mL of* E. coli* culture expressing each OfPPO was centrifuged at 10,000 g for 3 min. The resulting* E. coli* cells were washed using 10 mM Tris buffer (pH7.4) three times and then suspended in 30% ethanol for nonproteolytic activation of recombinant PPOs. After incubation at room temperature for 30 min, the reaction mixture was centrifuged at 10,000 g for 3 min. The cell pellet containing PPOs was then incubated with 250 *μ*L of dopamine (10 mM) for 5 min. Then PO activity of each sample was measured by monitoring* A*_490_ in a microplate reader (Bio-Tek instrument, Inc.). One unit of PO activity was defined as the amount of enzyme producing an increase in absorbance (Δ*A*_490_) of 0.001 per min.

## 3. Results

### 3.1. Cloning and Analysis of* O. furnacalis* PPO cDNAs

With “next generation” high-throughput sequencing, we obtained an* O. furnacalis* transcriptome dataset containing 62,382 unigenes [20]. Among these, three (1,299-bp CL5552.Contig1, 2,366-bp CL997.Contig1, and 1,153-bp Unigene28348) were predicted to encode potential PPOs [20]. The 2,366-bp CL997.Contig1 contains a complete 2,079-bp open reading frame and encodes a polypeptide sequence with 100% identity to* O. furnacalis* PPO (GenBank: ABC59699) previously reported by Feng et al. [37]. Here we designated it as* OfPPO2*. We performed 5′- and 3′-RACE to obtain the missing ends of the transcript CL5552.Contig1 and Unigene28348. After validation, CL5552.Contig1 was actually an assembly mosaic consisting of two variant sequences. The obtained full-length cDNA was nominated as* OfPPO1a*,* OfPPO1b*, and* OfPPO3*, respectively. They were submitted to NCBI and assigned the accession number as KX452359, KX437622, and KX437621, respectively.* OfPPO1a* and* OfPPO1b* matched with the 3′- and 5′-end of CL5552.Contig1, respectively (Figure 1(a)). We conducted PCR using the primers common in both* OfPPO1a* and* OfPPO1b* (Table  S1) and subjected the PCR products for sequencing. Among 12 clones randomly selected, 6 ones were identical to* OfPPO1a* while the other 6 were identical to* OfPPO1b*. This indicated that* OfPPO1a* and* OfPPO1b* were expressed with the same abundance in* O. furnacalis* larvae.

**Figure 2 fig2:**
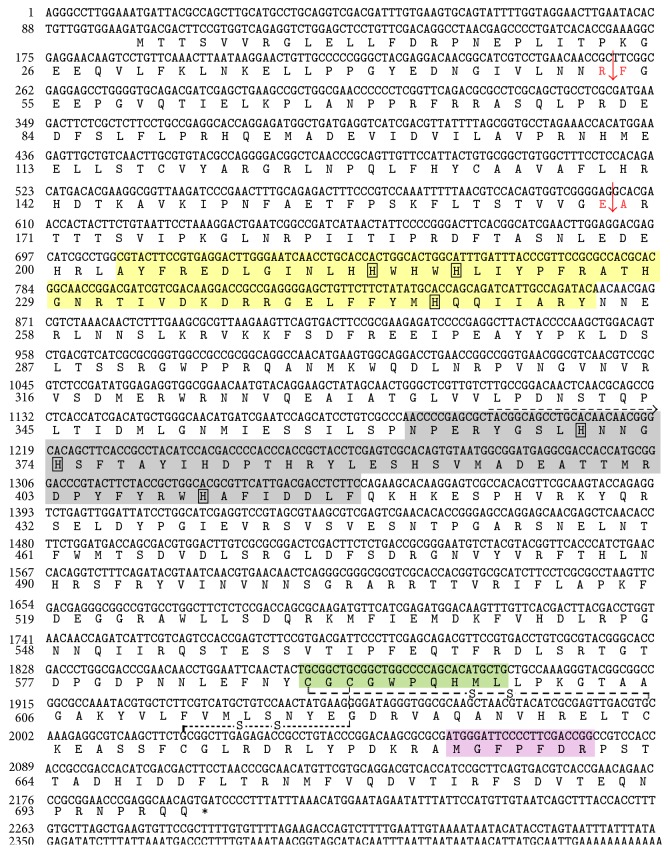
Nucleotide and amino acid sequence of* O. furnacalis PPO3*. The deduced amino acid sequences are shown below the nucleotide sequence of* PPO3* (NCBI accession number: KX437621). Nucleotides are numbered from the first base at the 5′-end. Amino acid residues were numbered from the initiating methionine. The one-letter code for each amino acid is aligned with the second nucleotide of the corresponding codon. The stop codon is marked with asterisk. The cDNA fragment from 3′-RACE started from the dashed-line arrow to the end. The predicted proteolytic cleavage bonds are shown in red and marked with red arrow. The potential copper binding site A (CuA), copper binding site B (CuB), the putative thiol ester sites, and a conserved motif at the C-terminus are shaded in yellow, grey, green, and purple, respectively. The six histidine residues absolutely conserved within the CuA and CuB are in the square boxes. Two predicted disulfide linkages are represented by dashed lines.

The full-length cDNA of OfPPO1a, OfPPO1b, and OfPPO3 contains 2,462, 2,391, and 2,388 nucleotides, with a 2,052-, 2,058-, and 2,100-bp open reading frame, respectively (Figures 1 and 2). A poly(A) tail was found in the cDNA sequences of OfPPO1b and OfPPO3, but not in OfPPO1a. The conceptual proteins deduced from nucleotide sequences of OfPPO1a, OfPPO1b, and OfPPO3 cDNA consist of 683, 685, and 699 amino acid residues, respectively (Figures 1 and 2). The OfPPO2 cDNA encodes a polypeptide with 692 amino acid residues [37]. All four* O. furnacalis* PPOs, including the previous OfPPO2, lack a typical secretion signal peptide at their N-terminal regions. The calculated molecular mass and isoelectric point of the deduced proteins are 78.6 kDa and 6.59 for PPO1a, 78.6 kDa and 5.99 for PPO1b, 79.8 kDa and 5.72 for PPO2, and 81.0 kDa and 7.41 for PPO3, respectively.

### 3.2. Sequence Comparisons and Phylogenetic Analysis of* O. furnacalis* PPOs

**Figure 3:**
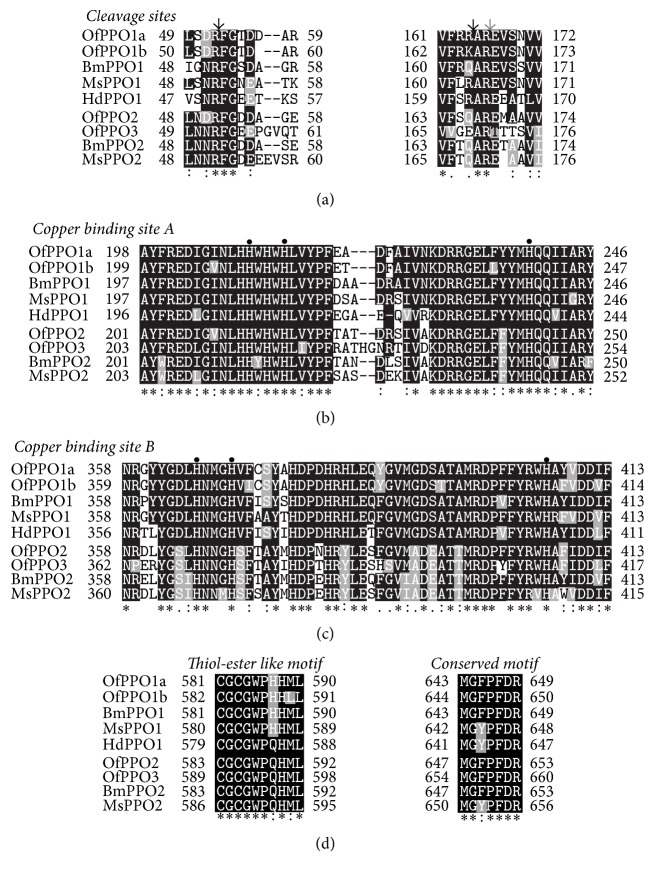
Multiple sequence alignment of conserved regions of insect PPOs. Amino acid sequences of PPOs from* O. furnacalis *PPO1a (OfPPO1a, KX452359), PPO1b (OfPPO1b, KX437622), PPO2 (OfPPO2, ABC59699), PPO3 (OfPPO3, KX437621),* B. mori* PPO1 (BmPPO1, AAG09304), PPO2 (BmPPO2, D49371),* M. sexta* PPO1 (MsPPO1, AF003253), PPO2 (MsPPO2, AAC37243), and* H. diomphalia* PPO1 (HdPPO1, AB079665) were aligned using the Clustal W. Alignments of potential cleavage sites (a), two independent copper binding sites (b and c), and two C-terminal conserved motifs (d) are shown. Numbers on both ends of each peptide represented amino acid residue numbers of the respective proteins. Amino acid residues shared by four or more proteins are shown by white letters on black background, while conservative amino acid substitutions are on grey background. Asterisks on the bottom of the alignment denoted amino acids shared by all proteins while small dots indicate conservatively substituted amino acids. The arrows in (a) indicate the potential proteolytic cleavage sites for PPO activation. Three large dots in (b) and (c) indicate the histidine residues coordinated with the copper atoms.

To clarify whether the amino acid residues and motifs critical for PPO activation and activity were also conserved in* O. furnacalis* PPOs, the deduced amino acid sequences of four* O. furnacalis* PPOs were aligned with well-characterized PPOs from* B. mori* [41],* M. sexta *[19, 42], and* H. diomphalia* [23, 24]. The alignments revealed that 6 key regions were highly conserved in 4* O. furnacalis* PPOs (Figure 3). The OfPPOs contain a conserved sequence (Arg-Phe-Gly) around the cleavage and activation site: ^52^RF^53^ in OfPPO1a, ^53^RF^54^ in OfPPO1b, ^51^RF^52^ in OfPPO2, and ^52^RF^53^ in OfPPO3 (Figure 3(a)). They possibly contain another proteolytic cleavage site: ^164^RA^165^ in OfPPO1a, ^165^KA^166^ in OfPPO1b, ^166^QA^167^ in OfPPO2, and ^168^EA^169^ in OfPPO3 (Figure 3(a)). Two copper binding sites, CuA and CuB, are very similar among these proteins: 198–246 and 358–413 in OfPPO1a, 199–247 and 359–414 in OfPPO1b, 201–250 and 358–413 in OfPPO2, and 203–254 and 362–417 in OfPPO3, respectively. These two copper binding sites carry three indispensable histidine residues in each site at H^210^-H^214^-H^239^ and H^266^-H^270^-H^406^ in OfPPO1a, H^211^-H^215^-H^240^ and H^267^-H^271^H^407^ in OfPPO1b, H^213^-H^217^-H^243^ and H^266^-H^270^-H^406^ in OfPPO2, and H^215^-H^219^-H^247^ and H^270^-H^274^-H^410^ in OfPPO3, respectively. These conserved histidine residues are likely to be involved in the copper binding (Figures 3(b) and 3(c)). Moreover, CuA contains hemocyanin encoding patterns like signature at ^209^HHWHWHLV^216^ in OfPPO1a, ^210^HHWHWHLV^217^ in OfPPO1b, ^212^HHWHWHLV^219^ in OfPPO2, and ^214^HHWHWHLI^221^ in OfPPO3, respectively. CuB contains both hemocyanin signatures (^395^TAMRDPFFY^403^ in OfPPO1a, ^396^TAMRDPFFY^404^ in OfPPO1b, ^395^TTMRDPFFY^403^ in OfPPO2, and ^399^TTMRDPYFY^407^ in OfPPO3) and tyrosinase signatures (^399^DPFFYRWHAYVD^410^ in OfPPO1a, ^400^DPFFYRWHAFVD^411^ in OfPPO1b, ^399^DPFFYRWHAFID^410^ in OfPPO2, and ^403^DPYFYRWHAFID^414^ in OfPPO3) (Figures 3(b) and 3(c)). Additionally, two conserved motifs are present in the C-terminal end of all 4 OfPPOs: one (CGCGWPH/QHML) matches the thiol ester region of *α*-macroglobulin and complement proteins C3 and C4, and the other one (MGFPFDR) is nearly identical in all compared PPOs (Figure 3(d)). The catalytic residue (E364) key to the hydroxylation and oxidation activities of* Anopheles gambiae* PPO8 [43] is identified as E^351^ in OfPPO1a, E^352^ in OfPPO1b, E^351^ in OfPPO2, E^355^ in OfPPO3, respectively. Besides, two disulfide bonds are predicted to be in the OfPPOs based on the comparisons with previously characterized* M. sexta* PPOs [42] and* A. gambiae* PPO8 [43]: Cys-581/Cys-623 and Cys-583/Cys-630 in OfPPO1a, Cys-582/Cys-624 and Cys-584/Cys-631 in OfPPO1b, Cys-583/Cys-627 and Cys-585/Cys-634 in OfPPO2, and Cys-589/Cys-634 and Cys-591/Cys-641 in OfPPO3 (Figures 1(b) and 2). All above results suggest that the obtained 4 nucleotide acid sequences (*OfPPO1a*-*OfPPO3*) encode functional PPO proteins.

**Figure 4:**
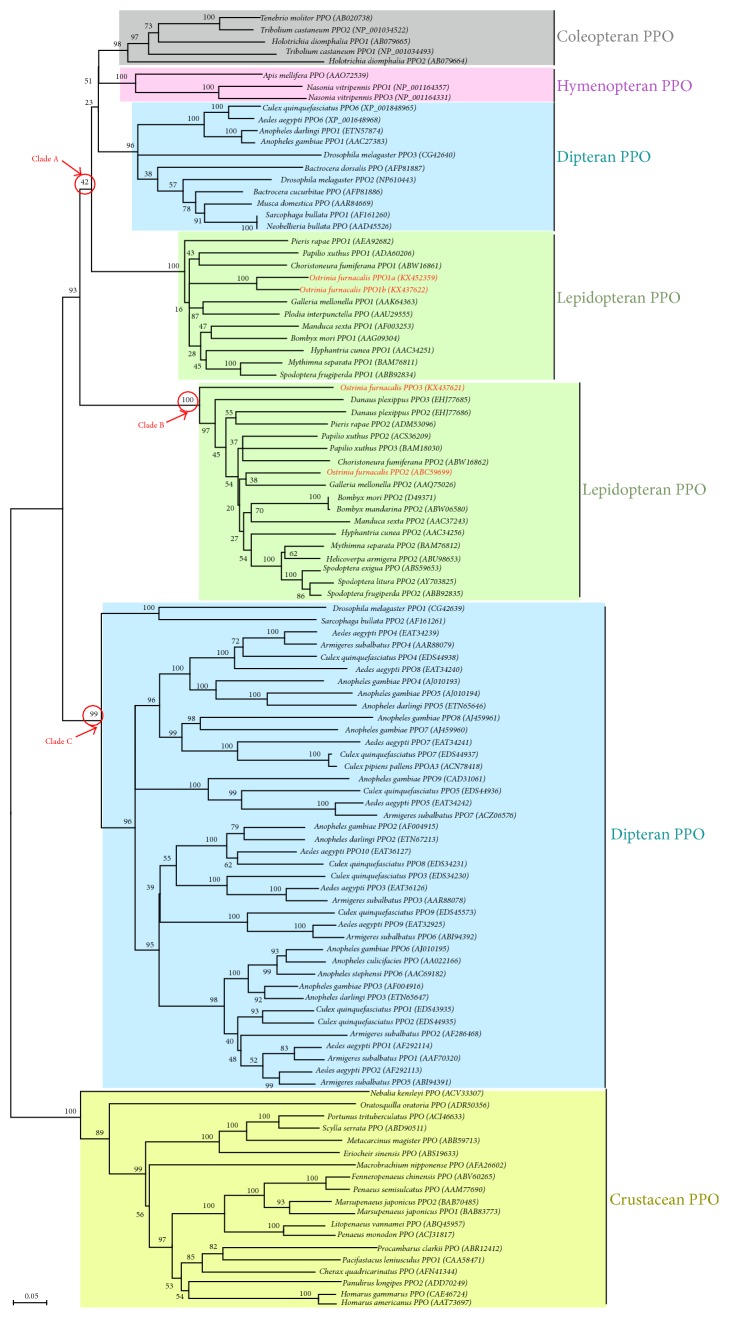
Phylogenetic analysis of* O. furnacalis *PPOs with other homologs. The used amino acid sequences of 108 PPOs were from 34 insect species and 18 crustacean species. The names of PPO genes were shown as scientific name of species followed by NCBI accession number of the gene. The* O. furnacalis* PPOs are marked in red. The branches specific for coleopteran, hymenopteran, dipteran, lepidopteran, and crustacean PPOs, respectively, are shaded in squares. Numbers at the nodes are bootstrap values as percentage. The circled bootstrap values indicate that the used insect PPOs were divided into three clades.

Database search and sequence alignment indicated that 4* O. furnacalis* PPOs exhibited high amino acid sequence similarity (42%–79% identity) with PPOs from other insect species: OfPPO1a, OfPPO1b, OfPPO2, and OfPPO3 are most similar in amino acid sequences to* B. mori* PPO1 (AAG09304),* Mythimna separata* PPO (BAM76811),* Galleria mellonella* PPO2 (AAQ75026), and* G. mellonella* PPO2 (AAQ75026), with the sequence identity of 74.5%, 74.1%, 79.3%, and 70.5%, respectively. A phylogenetic analysis was performed to investigate the evolutionary relationships between OfPPOs and other homologs. As shown in Figure 4, insect and crustacean PPOs form separate clades in the phylogenetic tree. The aligned insect PPOs (totally 89 PPOs from 34 insect species) are clustered into six distinct groups, including the subfamilies for two lepidopteran PPOs, two dipteran PPOs, one coleopteran PPO, and one hymenopteran PPO. The only inconsistency is Hemiptera PPO (*Choristoneura fumiferana* PPO2, ABW16862) is assigned to the group of lepidopteran PPOs. These six groups are divided into three major clades: Clade A consists of the conserved PPOs distributed among various insect orders, whereas Clades B and C comprise the paralogs specifically present in Lepidoptera and Diptera, respectively (Figure 4).* O. furnacalis* PPO1a and PPO1b clustered with lepidopteran PPOs in Clade A and OfPPO2 and OfPPO3 grouped with lepidopteran PPOs in Clade B.

### 3.3. Expression Profiles of 4 PPO Transcripts in* O. furnacalis*

**Figure 5:**
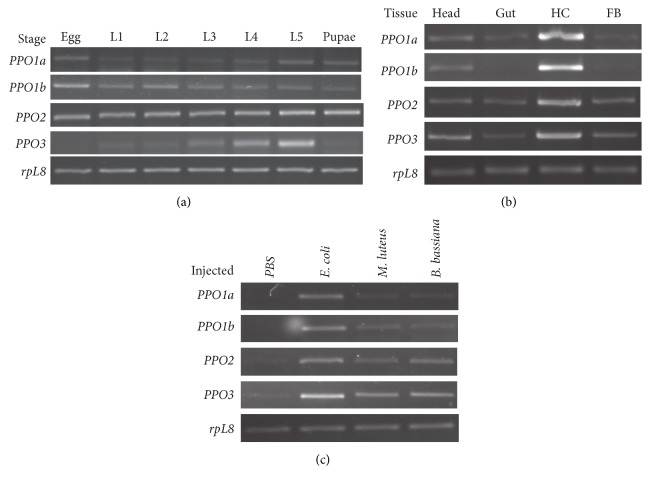
RT-PCR analysis of the expression of four* O. furnacalis PPOs*. (a) Expression profiles of* OfPPOs* at different stages of development. RNA was extracted from the whole bodies collected from eggs, first-instar (*L1*), second-instar (*L2*), third-instar (*L3*), fourth-instar (*L4*), fifth-instar (*L5*) larvae, and pupae. The* rpL8* was used as an internal control. (b) Expression patterns of* OfPPOs* in different tissues of* O. furnacalis* larvae. Tissues including head, gut, hemocytes (HC), and fat bodies (FB) were collected from day-zero, fifth-instar larvae for RNA extraction. RT-PCR was performed to assess the transcript level of* OfPPOs*. The* rpL8* was used to normalize the templates. (c) Expression profiles of* PPOs* in* O. furnacalis* larvae upon microbial challenge. Day 1, fifth-instar larvae were infected with water,* E. coli*,* M. luteus*, or* B. bassiana*. RNA was prepared from the whole bodies 24 h after injection. RT-PCR was used to analyze the transcript change of* OfPPOs*. The* rpL8* was used as an internal standard to indicate a consistent total mRNA amount.

We analyzed the mRNA levels of 4* O. furnacalis PPOs* in various development stages, different tissues, and different pathogen challenges using semiquantitative RT-PCR methods. As shown in Figure 5, the four* O. furnacalis PPOs* exhibited distinct expression patterns. The* OfPPO1a* transcript was at the highest levels in eggs and the fifth-instar larval stage and then in the pupae. This transcript was barely detectable in the first to fourth-instar larval stage, especially in the first and second-instar larval stage.* OfPPO1b* and* OfPPO2* were expressed in all tested developmental stages, including egg, larval, and pupal stages. However,* OfPPO1b* was expressed at the highest levels in eggs while* OfPPO2* was expressed at similar levels in all stages. The* OfPPO3* mRNA level increased from the second-instar larval stage, reached a maximum at the fifth-instar stage, and decreased dramatically in the pupal stage (Figure 5(a)). RT-PCR analyses showed that the 4* O. furnacalis PPO* mRNAs were all detected in the hemocytes at the highest levels among the tested four tissues including heads, guts, hemocytes, and fat bodies.* OfPPO1b* mRNA was not detected in guts and fat bodies (Figure 5(b)).

To check the* O. furnacalis PPO* expression patterns after exposure to microbial elicitors, we analyzed their transcript level after* O. furnacalis* larvae were injected with* E. coli*,* M. luteus*,* B. bassiana*, or water as a control. The result from RT-PCR assay indicated that all 4* O. furnacalis PPO* mRNA levels clearly increased in the larva challenged by bacteria or fungi, especially in the* E. coli*-injected larvae. Injection of* M. luteus* or* B. bassiana* resulted in a much smaller increase in the* OfPPO1a* transcript level compared to the treatment with* E. coli *(Figure 5(c)).

### 3.4. Production of Recombinant* O. furnacalis* PPOs in the Prokaryotic Expression System

As a first step to biochemically characterize* O. furnacalis* PPOs, we tried to produce recombinant proteins using prokaryotic expression system. After several preliminary experiments (data not shown), we selected the pET system for recombinant* O. furnacalis* PPO production, in which the inserted* O. furnacalis PPO* genes were under control of strong bacteriophage T7 transcription and translation signals and their expression was induced by providing a source of T7 RNA polymerase in the host BL21 (DE3) cells. Using a highly simplified and efficient PCR-based cloning technique [40], the complete open reading frame of 4* O. furnacalis PPOs* was successfully inserted into the pET28a vector individually. In the constructed plasmids, two 6 × His tags were added into the amino- and carboxyl-terminus of recombinant PPO proteins, respectively (Figure  S1). Additionally, we successfully transformed dual plasmids into BL21 (DE3) cells in order to express pairs of PPOs simultaneously, including OfPPO1a and OfPPO2, OfPPO1a and OfPPO3, OfPPO1b and OfPPO2, and OfPPO1b and OfPPO3 (Figure  S3A).

**Figure 6:**
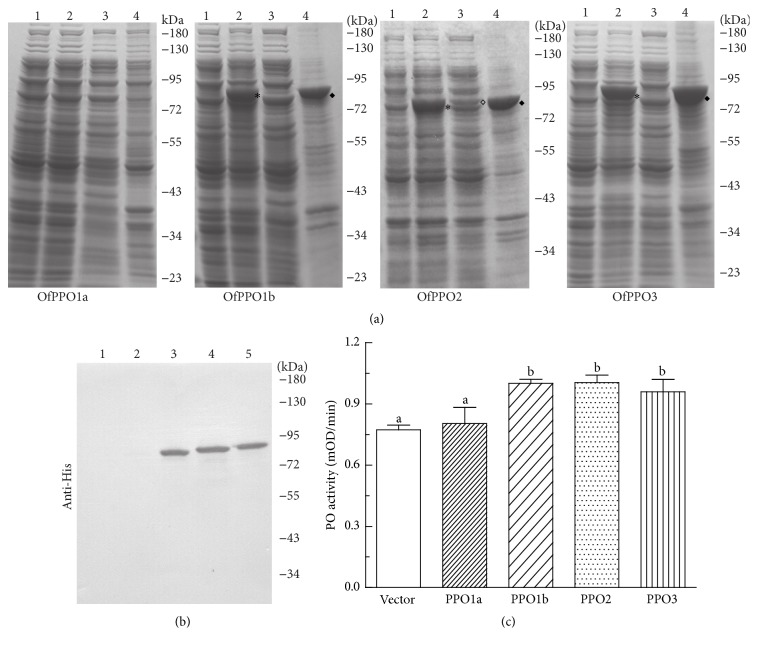
Recombinant expression of four* O. furnacalis* PPOs in* E. coli*. (a) SDS-PAGE analysis of recombinant PPOs. Four* O. furnacalis* PPOs were expressed in* E. coli* at 18°C for 12 h. The cultured cells were harvested and treated as described in Materials and Methods. The obtained protein samples were then subjected to 10% SDS-PAGE and visualized by Coomassie brilliant blue staining. Lane 1: 10 *μ*L of cell lysate collected before the addition of IPTG. Lane 2: 5 *μ*L of cell lysate collected 12 h after the addition of IPTG. Lane 3: 10 *μ*L of supernatant of cell lysate treated by sonication (soluble faction). Lane 4: 10 *μ*L of precipitate of cell lysate treated by sonication (insoluble fraction). The total recombinant OfPPO1b, OfPPO2, or OfPPO3 is indicated by the asterisk, while the insoluble protein is indicated by solid diamond. The soluble OfPPO2 is indicated by hollow diamond. (b) Western blot analysis of recombinant PPOs. The obtained samples in (a) were subjected to immunoblotting using mouse anti-His as primary antibodies. Lane 1: 1 *μ*L of cell lysate collected before the addition of IPTG. Lanes 2–5: 1 *μ*L of cell lysate collected 12 h after the addition of IPTG, which contained recombinant OfPPO1a, OfPPO1b, OfPPO2, and OfPPO3, respectively. (c) PO activity assay of recombinant PPOs. The cell lysate was prepared as described in “Materials and Methods and incubated with 30% ethanol for activation [30]. PO activity of each protein sample was measured with dopamine as substrate. The bars represent mean ± SD (*n* = 3). Bars labeled with different letters (a or b) are significantly different (one-way ANOVA, followed by the Newman-Keuls test, *p* < 0.05).

The recombinant expression of 4* O. furnacalis* PPOs was evaluated at different temperatures and induction conditions to determine the optimum parameters for protein expression (data not shown). Eventually, three of four* O. furnacalis* PPOs (OfPPO1b, OfPPO2, and OfPPO3, but not OfPPO1a) were successfully expressed under the IPTG induction (Figure 6(a)). Although the insect-produced PPOs are soluble proteins [24, 41, 42], large proportions of recombinant OfPPO1b, OfPPO2, and OfPPO3 formed insoluble aggregates in the* E. coli* cells even using a low concentration of IPTG (0.1 mM) and a relatively low temperature (18°C). These aggregates fractionated with the cell debris following lysis (Figure 6(a)). Under the same induction and lysis conditions, the solubility of recombinant OfPPO2 was obviously higher than that of recombinant OfPPO1b and OfPPO3. The soluble fractions of recombinant OfPPO1b and OfPPO3 were nearly undetectable in polyacrylamide gel electrophoresis (Figure 6(a)).

Additionally, recombinant OfPPO1b, OfPPO2, and OfPPO3, but not OfPPO1a, could be recognized by antibody against His-tag (Figure 6(b)). The cultured cells were treated with 30% ethanol for activating recombinant OfPPOs as described by Li et al. [30]. The resulting PO activity was checked using dopamine as substrate. As Figure 6(c) shown, four ethanol-treated OfPPOs with the exception of OfPPO1a exhibited detectable PO activities. There was no significant difference among the PO activity of recombinants OfPPO1b, OfPPO2, and OfPPO3.

## 4. Discussion

Phenoloxidase (PO) is the important enzyme in the melanin synthesis, a pathway in which some secondary reaction products including reactive oxygen species (ROS) and reactive nitrogen species (RNS) are also generated [3, 11]. PO plays a key role in multiple physiological processes including wound healing [44], hemolymph clotting [45], and innate immune response especially in defensive encapsulation and melanization of foreign organisms [11, 46]. In this study, we cloned full-length nucleotide sequences for 3 previously unknown* O. furnacalis PPOs* (*OfPPO1a*,* OfPPO1b*, and* OfPPO3*) together with* OfPPO2*, which had been reported previously [37]. We investigated the expression profiles of these 4 PPOs and performed the recombinant expression using the prokaryotic expression system.

The three previously undocumented full-length cDNA sequences for* O. furnacalis PPOs* encode polypeptides with ~700 amino acid residues and apparent molecular weight of ~80 kDa. As described in other insects [41, 47], the conceptual* O. furnacalis* PPO proteins also contain four conserved regions including two possible copper binding sites, one thiol ester-like motif and one common C-terminal end (Figures 1 [Fig fig2]–3). The copper binding sites with six absolutely conserved histidine residues are common in all arthropod POs and other closely related proteins such as arthropod hemocyanins [47]. They play an important role in encapsulation of foreign particles [48].* O. furnacalis* PPOs are potentially responsible for oxygen transport and melanization process due to the presence of these two binding sites. The high similarity and the shared conserved domains of* O. furnacalis* PPOs and arthropod PPOs infer that the identified* O. furnacalis* PPOs in this study are members of the PO family.

In addition to the structurally necessary domains and motifs mentioned above, there are other two aspects worthy of note in* O. furnacalis* PPOs. Firstly, no typical secretion signal peptide was predicted at the N-terminal region of the deduced* O. furnacalis* PPO1a, PPO1b, and PPO3 (Figures 1 and 2). This is also consistent with the case in* O. furnacalis* PPO2 [37] and other insect PPOs excluding venom PPO in the parasitoid wasp,* Pimpla hypochondriaca* [11, 19, 49]. In* D. melanogaster*, PPO was demonstrated to be released by the rupture of crystal cells [21]. In other arthropods, there are possibly other ways to release PPO than by cell rupture [11, 50]. So far, it is unclear whether and how* O. furnacalis* PPOs are released into the extracellular milieu. Secondly, the released PPO exists as zymogen in the hemolymph and must be activated by proteolytic cleavage [11, 20]. The presumed cleavage and activation sites of 4* O. furnacalis* PPOs were predicted between Arg^52^ and Phe^53^ in OfPPO1a, Arg^53^ and Phe^54^ in OfPPO1b, Arg^51^ and Phe^52^ in OfPPO2, and Arg^52^ and Phe^53^ in OfPPO3, as previously reported [13, 16, 46]. In* H. diomphalia*, HdPPO was cleaved at the conserved bond Arg^50^-Phe^51^, followed by the second cleavage at Arg^162^-Ala^163^ [23, 24]. In the housefly* Musca domestica*, the activation of PPO may occur after the cleavage at Arg^164^-Glu^165^ [51]. The Arg-Ala bond in HdPPO was identified at the common position in OfPPO1a and OfPPO1b, but not in OfPPO2 and OfPPO3. The Arg-Glu bond in MdPPO was consistently present in* O. furnacalis* PPOs except for OfPPO3 with Arg-Thr instead (Figure 3(a)). We have determined two serine proteases, SP13 and SP105, which functioned as prophenoloxidase-activating proteases to cleave and activate OfPPO2 [52]. However, the precise activation site(s) of* O. furnacalis* PPOs is still unknown. It is also unclear whether or not there exist other possible cleavage sites other than the typical Arg-Phe bond.

Insect POs are artificially divided into tyrosinase-type POs and laccase-type POs in some reports: the former can hydroxylate monophenols to* o*-diphenols (EC 1.14.18.1) and then oxidize the* o*-diphenols to quinones (EC 1.10.3.1), but it cannot oxidize* p*-diphenols; the latter lack the monophenol monooxygenase activity but can oxidize both* o*- and* p*-diphenols (EC 1.10.3.2) [53, 54]. A phylogenetic analysis of the 4* O. furnacalis* PPOs with insect tyrosinase-type and laccase-type POs revealed two distinct clades with 100% bootstrap support. All 4* O. furnacalis* PPOs fell inside the cluster of tyrosinase-type POs (Figure  S2). This suggests that the identified* O. furnacalis* PPOs are orthologous to tyrosinases rather than laccases. The precise classification of* O. furnacalis* PPOs cannot be determined until their activities toward monophenols and* p*-diphenols are analyzed. Furthermore, the phylogenetic relationship among* O. furnacalis* PPOs and other arthropod tyrosinase-type POs was proposed in this work. Lepidopteran, dipteran, coleopteran, and hymenopteran PPOs were separated in distinct clusters. Lepidopteran and dipteran PPOs each fell into two separate groups.* O. furnacalis* PPO1a and PPO1b were clustered in one lepidopteran PPO group while PPO2 and PPO3 were in the other lepidopteran PPO group (Figure 4). It suggests that lepidopteran* PPO* genes including* O. furnacalis PPOs* may have undergone duplication and divergence during evolution.

It is of particular interest that up to 4* O. furnacalis PPO* sequences are identified in this study since only two* PPO* genes have been reported in most lepidopteran species [13, 16]. These 4* O. furnacalis PPOs* were differentially expressed with respect to developmental stages, tissues, or induced conditions (Figure 5). Even* PPO1a* and* PPO1b*, which are 86.82% identical in deduced amino acid sequences, also exhibited distinct expression patterns. For example,* PPO1a* mRNAs were detected in all tested tissues including head, gut, hemocytes, and fat bodies while* OfPPO1b* transcripts were barely detectable in gut and fat bodies (Figure 5(b)). Moreover, recombinant OfPPO1b, OfPPO2, and OfPPO3 could be produced successfully in prokaryotic expression system and be activated by ethanol. OfPPO1a failed to be expressed in* E. coli* (Figure 6). Three* PPO* genes were also identified in the swallowtail butterfly,* Papilio xuthus* (Figure 4) [55]. Therefore, we infer that all identified* PPOs* indeed exist and potentially perform different functions in* O. furnacalis*, although we currently do not know why* O. furnacalis* possesses more* PPO* genes than other lepidopterans. Explanations for the presence of 4* PPO* sequences will be possible only when more data on PO function in* O. furnacalis* are obtained. Obtaining the recombinant PPO proteins* in vitro* in this study was just the first step to this aim. The 3* O. furnacalis* PPOs expressed in* E. coli* were observed to have obvious enzyme activities after activation by ethanol (Figure 6(c)). In order to potentially increase the solubility, we also tried to obtain PPO heterodimer by performing the coexpression, but failed possibly due to plasmid instability or incompatibility (Figure  S3). In future work, we will attempt to produce the recombinant PPOs with higher activity and then investigate their activation mechanisms and detailed functions.

## Supplementary Material

The following supplementary material is available: Table S1: Oligonucleotides used for cloning, RT-PCR, and plasmid construction and confirmation. Figure S1: Amino acid sequences of 4 recombinant O. furnacalis PPOs expressed in E. coli. The amino acid sequences were deduced from the nucleotide sequences of the insert in constructed plasmids via FastCloning. The amino acid residues from the expression vector pET28a were underlined. Figure S2: Phylogenetic analysis of O. furnacalis PPOs with other insect tyrosinase- and laccase-type PPOs. The analysis was performed using the MEGA 5 program. The consensus tree was constructed using the neighbor-joining method. Gaps were treated as characters and statistical analysis was performed by the bootstrap method with 1000 repetitions. Only bootstrap values greater than 90 were shown. The names of used genes were shown as scientific name of species followed by NCBI accession number of this specific gene. The branches specific for tyrosinase- and laccase-type PPOs were shaded in yellow and blue, respectively. The circled bootstrap values indicated that O. furnacalis PPOs belong to tyrosinase-type PPOs. Figure S3: Co-expression of O. furnacalis PPOs in E. coli. (A) Confirmation of dual plasmid transformation by PCR. Plasmids encoding OfPPO1a or OfPPO1b were respectively transformed to E. coli BL21 (DE3) cells which already contained plasmids encoding OfPPO2 or OfPPO3. After selection by kanamycin, a single clone was used as template for PCR using two pairs of primers listed in Table S1. The PCR products were separated by electrophoresis on a 1.0% agarose gel. (B) SDS-PAGE analysis of recombinant PPOs. O. furnacalis PPOs were expressed in E. coli at 18°C for 12 h. The cultured cells were harvested and treated as described in “Materials and Methods”. The obtained protein samples were then subjected to 10% SDS-PAGE and visualized by Coomassie brilliant blue staining. Lane 1 : 10 μL of cell lysate collected before the addition of IPTG. Lane 2–5 : 5 μL of cell lysate collected 12 h after the addition of IPTG, which contained recombinant OfPPO1a, OfPPO1b, OfPPO2, and OfPPO3, respectively. Lane 6 and 7 : 10 μL of cell lysate simultaneously expressing recombinant OfPPO1a and OfPPO2 (OfPPO1a/2), collected before or after the addition of IPTG, respectively. Lane 8 and 9 : 10 μL of cell lysate simultaneously expressing recombinant OfPPO1a and OfPPO3 (OfPPO1a/3), collected before or after the addition of IPTG, respectively. Lane 10 and 11 : 10 μL of cell lysate simultaneously expressing recombinant OfPPO1b and OfPPO2 (OfPPO1b/2), collected before or after the addition of IPTG, respectively. Lane 12 and 13 : 10 μL of cell lysate simultaneously expressing recombinant OfPPO1b and OfPPO3 (OfPPO1b/3), collected before or after the addition of IPTG, respectively. (C) Western blot analysis of recombinant PPOs. The obtained samples in (B) were subjected to immunoblotting using mouse anti-His as primary antibodies. Lane 1 : 20 μL of cell lysate collected before the addition of IPTG. Lane 2 : 10 μL of cell lysate collected 12 h after the addition of IPTG. Lane 3 : 20 of supernatant of cell lysate treated by sonication (soluble faction). Lane 4 : 20 μL of precipitate of cell lysate treated by sonication (insoluble fraction).
