# Supplementary material for: Cloning, Expression, and Characterization of Prophenoloxidases from Asian Corn Borer, Ostrinia furnacalis (Gunée)
Source: J Immunol Res. 2016 Dec 18;2016:1781803. doi: 10.1155/2016/1781803 (PMC5203920; doi:10.1155/2016/1781803)
Supplement: Supplementary file 1 — The following supplementary material is available: Table S1: Oligonucleotides used for cloning, RT-PCR, and plasmid construction and confirmation. Figure S1: Amino acid sequences of 4 recombinant O. furnacalis PPOs expressed in E. coli. The amino acid sequences were deduced from the nucleotide sequences of the insert in constructed plasmids via FastCloning. The amino acid residues from the expression vector pET28a were underlined. Figure S2: Phylogenetic analysis of O. furnacalis PPOs with other insect tyrosinase- and laccase-type PPOs. The analysis was performed using the MEGA 5 program. The consensus tree was constructed using the neighbor-joining method. Gaps were treated as characters and statistical analysis was performed by the bootstrap method with 1000 repetitions. Only bootstrap values greater than 90 were shown. The names of used genes were shown as scientific name of species followed by NCBI accession number of this specific gene. The branches specific for tyrosinase- and laccase-type PPOs were shaded in yellow and blue, respectively. The circled bootstrap values indicated that O. furnacalis PPOs belong to tyrosinase-type PPOs. Figure S3: Co-expression of O. furnacalis PPOs in E. coli. (A) Confirmation of dual plasmid transformation by PCR. Plasmids encoding OfPPO1a or OfPPO1b were respectively transformed to E. coli BL21 (DE3) cells which already contained plasmids encoding OfPPO2 or OfPPO3. After selection by kanamycin, a single clone was used as template for PCR using two pairs of primers listed in Table S1. The PCR products were separated by electrophoresis on a 1.0% agarose gel. (B) SDS-PAGE analysis of recombinant PPOs. O. furnacalis PPOs were expressed in E. coli at 18°C for 12 h. The cultured cells were harvested and treated as described in “Materials and Methods”. The obtained protein samples were then subjected to 10% SDS-PAGE and visualized by Coomassie brilliant blue staining. Lane 1 : 10 μL of cell lysate collected before the addition of IPTG [file 1781803.f1.pdf]

Supporting information:

Cloning, expression, and characterization of prophenoloxidase from Asian corn borer, *Ostrinia furnacalis* (Gunée)

Shasha Zhang[&], Fang Hong[&], He Song, Lei Wang, Qizhi Liu, Chunju An

The following supplementary material is available:

Table S1: Oligonucleotides used for cloning, RT-PCR, and plasmid construction and confirmation.

Figure S1: Amino acid sequences of 4 recombinant *O. furnacalis* PPOs expressed in *E. coli*. The amino acid sequences were deduced from the nucleotide sequences of the insert in constructed plasmids via FastCloning. The amino acid residues from the expression vector pET28a were underlined.

Figure S2: Phylogenetic analysis of *O. furnacalis* PPOs with other insect tyrosinase- and laccase-type PPOs. The analysis was performed using the MEGA 5 program. The consensus tree was constructed using the neighbor-joining method. Gaps were treated as characters and statistical analysis was performed by the bootstrap method with 1000 repetitions. Only bootstrap values greater than 90 were shown. The names of used genes were shown as scientific name of species followed by NCBI accession number of this specific gene. The branches specific for tyrosinase- and laccase- type PPOs were shaded in yellow and blue, respectively. The circled bootstrap values indicated that *O. furnacalis* PPOs belong to tyrosinase-type PPOs.

Figure S3: Co-expression of *O. furnacalis* PPOs in *E. coli*. (A) Confirmation of dual plasmid transformation by PCR. Plasmids encoding OfPPO1a or OfPPO1b were respectively transformed to *E. coli* BL21 (DE3) cells which already contained plasmids encoding OfPPO2 or OfPPO3. After selection by kanamycin, a single clone was used as template for PCR using two pairs of primers listed in Table S1. The PCR products were separated by electrophoresis on a 1.0% agarose gel. (B) SDS-PAGE analysis of recombinant PPOs. *O. furnacalis* PPOs were expressed in *E. coli* at 18 ºC for 12 h. The cultured cells were harvested and treated as described in "Materials and Methods". The obtained protein samples were then subjected to 10% SDS-PAGE and visualized by Coomassie brilliant blue staining. Lane 1: 10 μL of cell lysate collected before the addition of IPTG. Lane 2-5: 5 μL of cell lysate collected 12 h after the addition of IPTG, which contained recombinant

OfPPO1a, OfPPO1b, OfPPO2, and OfPPO3, respectively. Lane 6 and 7: 10 μL of cell lysate simultaneously expressing recombinant OfPPO1a and OfPPO2 (OfPPO1a/2), collected before or after the addition of IPTG, respectively. Lane 8 and 9: 10 μL of cell lysate simultaneously expressing recombinant OfPPO1a and OfPPO3 (OfPPO1a/3), collected before or after the addition of IPTG, respectively. Lane 10 and 11: 10 μL of cell lysate simultaneously expressing recombinant OfPPO1b and OfPPO2 (OfPPO1b/2), collected before or after the addition of IPTG, respectively. Lane 12 and 13: 10 μL of cell lysate simultaneously expressing recombinant OfPPO1b and OfPPO3 (OfPPO1b/3), collected before or after the addition of IPTG, respectively. (C) Western blot analysis of recombinant PPOs. The obtained samples in (B) were subjected to immunoblotting using mouse anti-His as primary antibodies. Lane 1: 20 μL of cell lysate collected before the addition of IPTG. Lane 2: 10 μL of cell lysate collected 12 h after the addition of IPTG. Lane 3: 20 of supernatant of cell lysate treated by sonication (soluble faction). Lane 4: 20 μL of precipitate of cell lysate treated by sonication (insoluble fraction).

Table S1 Oligonucleotides used for cloning, RT-PCR, and plasmid construction

Name	Forward primers (5′-3′)	Reverse primers (5′-3′)
**Cloning of PPO1**		
F1	GAGGCTTACTTCCCCAAACTG	
F2	AACACGGCTTGCTAACAACT	
F3	AAATGGCAGACGCAGAAGC	
R1		TGAGACATACCTACATAGAAG
R2		TAACGGTACATTGGTATCTGCT
R3		CCAGGCACCAGTACAGCGTT
F4	AGTTTGGATACCTGGATTAGT	
F5	CGACCTGCATAAGACCAAG	
R5		GCATGGGCACGCTCAGGTC
R6		TCTCCTTCATTGCCAGTC
**Cloning of PPO3**		
F6	GCATCGTCCTGAACAACC	
F7	TGTGAAGTGCAGTATTTTGG	
R7		CGCGGATCCTCCACTAGTGATTTCACTATAGG
**Simultaneous amplification of both PPO1a and PPO1b**		
PPO1a&b	CAGCTGCCTTACAACGAG	GTGAAGTCGAGTCCCCTG
**RT-PCR**		
OfPPO1a	CAAGGTTAAGCTGCCGAAGTAT	TTCGTGCGTAAGGTGGGT
OfPPO1b	CGACCTGCATAAGACCAAG	GTCGTGCTGAAGATGAGT
OfPPO2	AGCAATGGAGGACTAACA	ACGCCGAAAGATTCAAGGT
OfPPO3	CATGCTGCTGCCAAAGGG	GCGGGTTCTGTTCGGTGA
rpL8	AAGCGAGGAACATCAGCC	GGTCTTGCCACCACGAAT
**Plasmid construction for recombinant production in *E.coli***		

PPO1a	CATCATCATCATCACGCAGACGAAGCCAGAAG<br>The sequence overlapping with the 3'-end of PCR-amplified vector is underlined.	GTGGTGGTGGTGGTGCTGCTGCGGCCGGAT<br>The sequence overlapping with the 5'-end of PCR-amplified vector is underlined.
PPO1b	CATCATCATCATCACGCAGACGCAGAAGCCA<br>The sequence overlapping with the 3'-end of PCR-amplified vector is underlined.	GTGGTGGTGGTGGTGCCGGCGCTGCTGCCTG<br>The sequence overlapping with the 5'-end of PCR-amplified vector is underlined.
PPO2	CATCATCATCATCACGCGGACGTTGTGAAAAG<br>The sequence overlapping with the 3'-end of PCR-amplified vector is underlined.	GTGGTGGTGGTGGTGCTGAGTGGGGTTCCTG<br>The sequence overlapping with the 5'-end of PCR-amplified vector is underlined.
PPO3	CATCATCATCATCACACGACTTCCGTGGTCAG<br>The sequence overlapping with the 3'-end of PCR-amplified vector is underlined.	GTGGTGGTGGTGGTGCTGTTGCCTCGGGTT<br>The sequence overlapping with the 5'-end of PCR-amplified vector is underlined.
pET28a	CACCACCACCACCACCACTGAGAT<br>The sequence complementary to the specific reverse primer is underlined.	GTGATGATGATGATGATGGCTGCT<br>The sequence complementary to the specific forward primer is underlined.

Figure S1

>OfPPO1a
MGSSHHHHHHADEARRNLMLFFDRPTEPCFMQKGDDKTVFQLPDHYYPDKYKALSTTLSDRFGTDDARIVQVSNIGLPDLSLPKQLPYNEQFSLFI
QKHREMAGSLIDTLVGMRNIEDLTSVCSYCQLHINPYMFNYCLAVALLHRDDTKGFNVPTVVQTFPDKFMDPKVFRRAREVSNVVTTGPRMPVVIE
QNYTASDAEPEQRVAYFREDIGINLHHWHWHLVYPFEADFAIVNKDRRGELFYYMHQQIIARYNVERFCNGLGRVERYTNFRAPIAEAYFPKLDSQ
VASRAWPPRFAGSILRDLDRPVDRIRIEVSELERWRDRFLQAIEENAVLVPGNRKVPLTEETGIDVLGNLMESSILSRNRGYYGDLHNMGHVFCSY
AHDPDHRHLEQYGVMGDSATAMRDPFFYRWHAYVDDIFNMHKVKLPKYGADRLDFPGIRVSSISVEGPAGRNTFGTQWEQSTVDLARGLDFTPRGS
VLAQFTHLTHEEFTYVIEVNNSSGRSTTGMFRIFIAPVNDDRGQPLSFADQRRLFIELDKFSQPLNTGNNTVRRSSVDSSVTIPYERTFMDQTKRP
GDPGSTTAAEFDFCGCGWPHHMLIPKGTARGYPMVLFVMVSNWNDDRILQDTSGSCNDAASYCGLRDRKYPDRRAMGFPFDRPPQASTLSSFLRPN
MAIRPCSVRFTDQVRIRPQQHHHHHH

>OfPPO1b
MGSSHHHHHHADAEAKKNLLLFFDRPTEPCFMQKGDDKTVFQLPEHYYPDKYRALSTTLSDRFGTDDARVVPVANIGLPDLSVPMQLPYNEQFSLF
IQKHREMAGNLIDTFVGMRNVEDLTSLCTYCQLRINPYMFNYCLSVALLHRDDTKGFNVPTLVQTFPDKFMDPKVFRKAREVSNVVTTGSRMPVEI
PTNLTTSELEPEQRVAYFREDIGVNLHHWHWHLVYPFETDFAIVNKDRRGELLYYMHQQIIARYTSERFCNGLGRVVRYTNFRAPIEEAYFPKLDS
QVASRAWPPRFAGSVLRDLDRPVDRIRIEVSELERWRDRVLQAIEENAVIVSGNRKVPLTEETGIDVLGNLMESSILSPNRGYYGDLHNMGHVICS
YAHDPDHRHLEQYGVMGDSTTAMRDPFFYRWHAFVDDVFDLHKTKLPKYGNDRLDFPGIRVSSISVEGPAGSNTFGTQWEQSMVDLARGLDFTPRG
SVLAKFTHLQHDEFTYVIEVNNTSGAAAMGMFRIFLAPVNDESGQPLRFEEQRRLFIELDKFSQPLNAGNNTIRRNSVDSSVTIPYERTFMDQSAR
PGDPGSAAAAEFDFCGCGWPHHLLIPKGTPQGFPMVLFVMVSNWNDDRVEQDTVGTCNDAASYCGIRDRKYPDKRAMGFPFDRPPQESTLSDFLRP
NMSIRQCTVKFTDATRIRQQRRHHHHHH

>OfPPO2
MGSSHHHHHHADVVKSFELLFDRPNEPLITPKGDNNALFQLTEQLLPEDYKNNGIELNDRFGDDAGERIPLQNLSQVPQFRVASQLPTDADFSLFL
PRHQEMATEVIDVLMNVPENQLQELLSTCVFARTNLNPQLFNYCYSVAIMHRRDTRNVKIPSFAETFPSKFLDSQVFSQAREMAAVVPQNIPRIPV
IIPRDFTASDLESEHRLAYFREDVGVNLHHWHWHLVYPFTASDRSIVAKDRRGELFFYMHQQIIARYNCERLNNNLKRVEKFSNWREPIPEAYFPK

LDSLTSSRGWPPRQSNMTWRDLDRPVDGLKIGVRDMEQWRTNIEEAIATGMARLPNGSTVDLNIDLLGNMMEASILSPNRDLYGSLHNNGHSFTAY
MHDPNHRYLESFGVMADEATTMRDPFFYRWHAFIDDIFQKHKESTFVRPYSNSELGNPGVRVMSAAIETQGGNQNELSTFWMSSDVDLSRGLDFSN
RGPVYARFTHLNHRPFQYVINVNNTGNARRTTVRIFISPKVDERNQPWILSDQRKMFIEMDRFVTPLNAGQNRIVRQSTESTFTIPFEQTFRDLSV
QGEDPRQVDLAAFNFCGCGWPQHMLVPKGSEAGSPYVLFVMLSNYEFDAVERTDGRQATCKEASSFCGIRDSLYPDKRAMGFPFDRPSNTATSIED
FIQPNMFLQDINIRLQCTPIVPNPRNPTQHHHHHH

>OfPPO3

MGSSHHHHHHTTSVVRGLELLFDRPNEPLITPKGEEQVLFKLNKELLPPGYEDNGIVLNNRFGEEPGVQTIELKPLANPPRFRRASQLPRDEDFSL
FLPRHQEMADEVIDVILAVPRNHMEELLSTCVYARGRLNPQLFHYCAAVAFLHRHDTKAVKIPNFAETFPSKFLTSTVVGEARTTTSVIPKGLNRP
IITIPRDFTASNLEDEHRLAYFREDLGINLHHWHWHLIYPFRATHGNRTIVDKDRRGELFFYMHQQIIARYNNERLNNSLKRAKKFSDFREEIPEA
YYPKLDSLTSSRGWPPRQANMKWQDLNRPVNGVNVRVSDMERWRNNVQEAIATGLVVLPDNSTQPLTIDMLGNMIESSILSPNPERYGSLHNNGHS
FTAYIHDPTHRYLESHSVMADEATTMRDPYFYRWHAFIDDLFQKHKESPHVRKYQRSELDYPGIEVRSVSVESNTPGARSNELNTFWMTSDVDLSR
GLDFSDRGNVYVRFTHLNHRSFRYVINVNNSGRARRTTVRIFLAPKFDEGGRAWLLSDQRKMFIEMDKFVHDLRPGNNQIIRQSTESSVTIPFEQT
FRDLSRTGTDPGDPNNLEFNYCGCGWPQHMLLPKGTAAGAKYVLFVMLSNYEGDRVAQANVHRELTCKEASSFCGLRDRLYPDKRAMGFPFDRPST
TADHIDDFLTRNMFVQDVTIRFSDVTEQNPRNPRQQHHHHHH

Figure S2

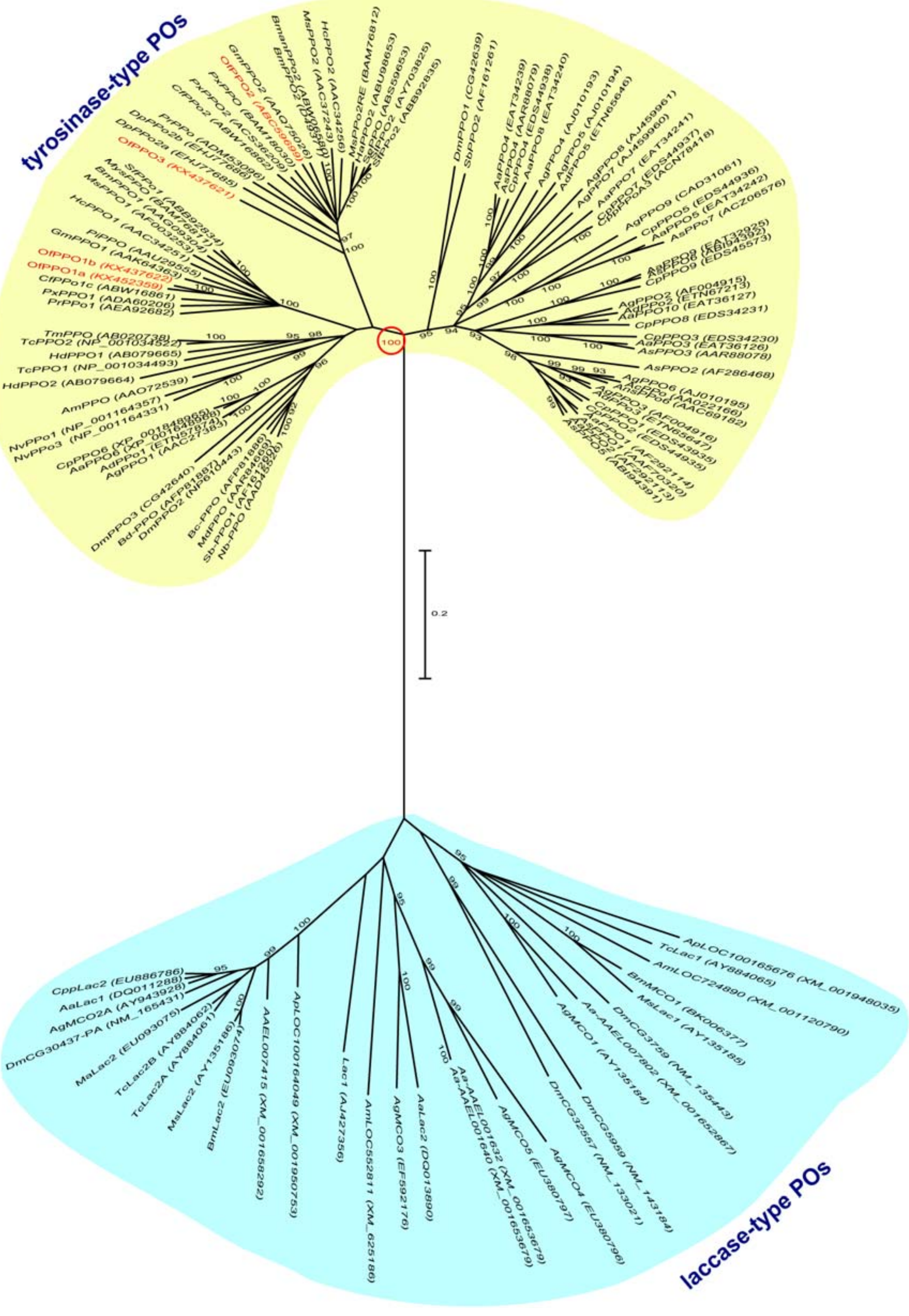

Figure S3

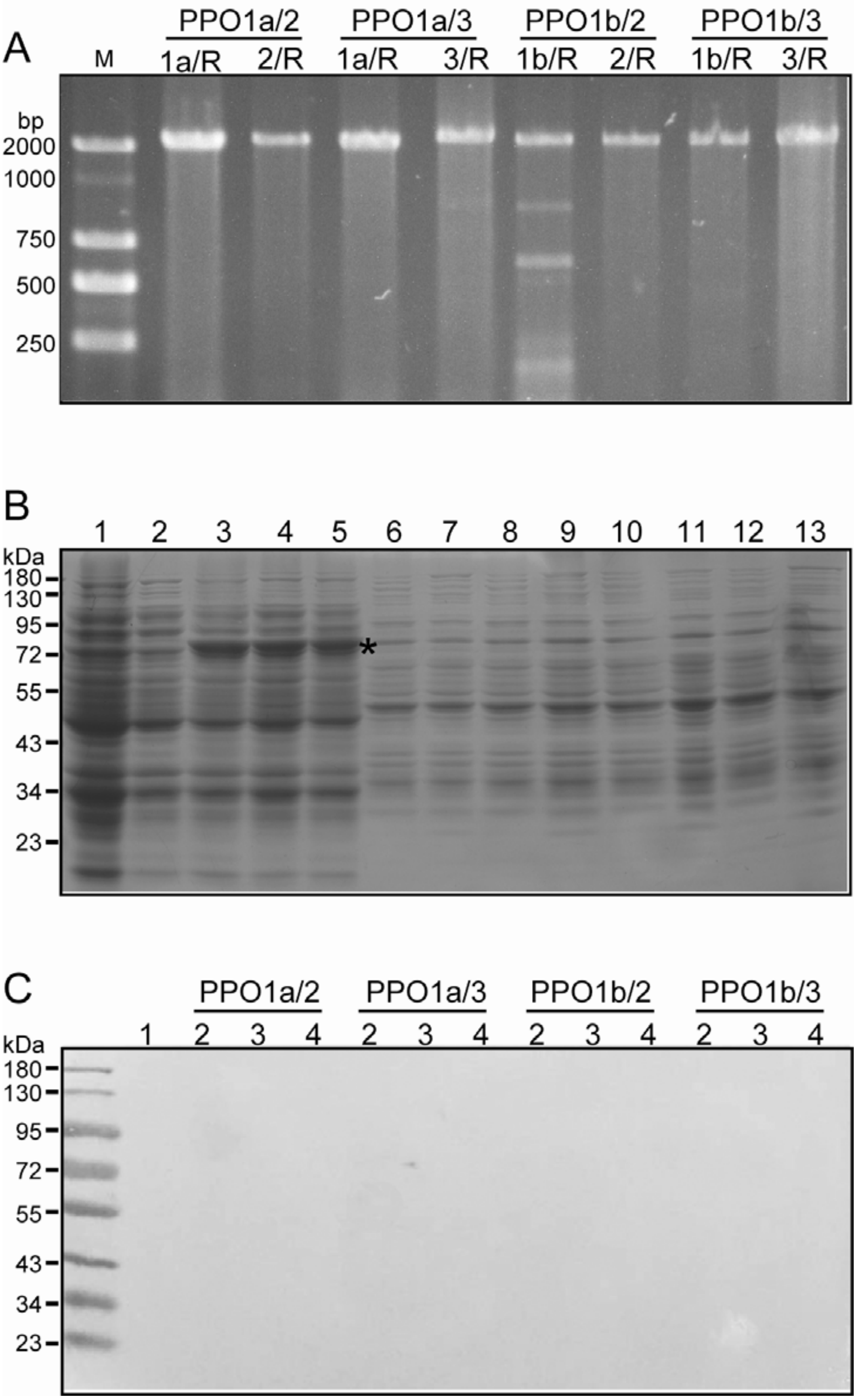
A
M
PPO1a/2
PPO1a/3
PPO1b/2
PPO1b/3
1a/R 2/R 1a/R 3/R 1b/R 2/R 1b/R 3/R
bp
2000
1000
750
500
250
B
1 2 3 4 5 6 7 8 9 10 11 12 13
kDa
180
130
95
72
55
43
34
23
*
C
PPO1a/2
PPO1a/3
PPO1b/2
PPO1b/3
1 2 3 4 2 3 4 2 3 4 2 3 4
kDa
180
130
95
72
55
43
34
23
